# The *Borrelia afzelii* outer membrane protein BAPKO_0422 binds human factor-H and is predicted to form a membrane-spanning β-barrel

**DOI:** 10.1042/BSR20150095

**Published:** 2015-08-18

**Authors:** Adam Dyer, Gemma Brown, Lenka Stejskal, Peter R. Laity, Richard J. Bingham

**Affiliations:** * Department of Biological Sciences, School of Applied Sciences, University of Huddersfield, Queensgate, Huddersfield HD1 3DH, U.K.; † Present Address: Department of Materials Science and Engineering, Sir Robert Hadfield Building, Mappin Street, University of Sheffield, Sheffield S1 3JD, U.K.

**Keywords:** β-barrel, *Borrelia*, Lyme disease, outer membrane protein A (OmpA), small angle X-ray scattering (SAXS), spirochete

## Abstract

The deep evolutionary history of the *Spirochetes* places their branch point early in the evolution of the diderms, before the divergence of the present day *Proteobacteria*. As a *spirochete*, the morphology of the *Borrelia* cell envelope shares characteristics of both Gram-positive and Gram-negative bacteria. A thin layer of peptidoglycan, tightly associated with the cytoplasmic membrane, is surrounded by a more labile outer membrane (OM). This OM is rich in lipoproteins but with few known integral membrane proteins. The outer membrane protein A (OmpA) domain is an eight-stranded membrane-spanning β-barrel, highly conserved among the *Proteobacteria* but so far unknown in the *Spirochetes*. In the present work, we describe the identification of four novel OmpA-like β-barrels from *Borrelia afzelii*, the most common cause of erythema migrans (EM) rash in Europe. Structural characterization of one these proteins (BAPKO_0422) by SAXS and CD indicate a compact globular structure rich in β-strand consistent with a monomeric β-barrel. *Ab initio* molecular envelopes calculated from the scattering profile are consistent with homology models and demonstrate that BAPKO_0422 adopts a peanut shape with dimensions 25×45 Å (1 Å=0.1 nm). Deviations from the standard C-terminal signature sequence are apparent; in particular the C-terminal phenylalanine residue commonly found in *Proteobacterial* OM proteins is replaced by isoleucine/leucine or asparagine. BAPKO_0422 is demonstrated to bind human factor H (fH) and therefore may contribute to immune evasion by inhibition of the complement response. Encoded by chromosomal genes, these proteins are highly conserved between *Borrelia* subspecies and may be of diagnostic or therapeutic value.

## Abstract (precis)

We present the characterization of BAPKO_0422, an outer membrane protein from *Borrelia afzelii*. Circular dichroism, size exclusion and small angle X-ray scattering data indicate a monomeric structure rich in β-strand. Recombinant BAPKO_0422 binds to human factor H.

## INTRODUCTION

Various species in the genus *Borrelia* are capable of zoonotic infection in humans when transmitted via the saliva of haematophagous ticks resulting in Lyme disease [1]). Considerable research has been directed at *Borrelia burgdorferi sensu stricto*, the most prevalent strain in North America but only a minor contributor to incidence rates in Europe and Asia. The available data on the natural hosts/vectors present in Europe show that the most common species present are *Borrelia garinii* and *Borrelia afzelii*, the latter of which is responsible for the vast majority of observed erythema migrans (EM) rash [2,3]. Although early treatment with antibiotics can be effective, symptoms may continue post-treatment probably due to a variety factors including the presence of immunogenic cell debris, survival of viable spirochetes [4–6] or the presence of persister phenotypes [7]. *Borrelia* have evolved numerous strategies to aid survival for extended periods within the mammalian host. Evasion strategies include antigenic variation [8,9], variable expression of surface antigens [10–12], invasion of immune privileged sites [13,14] and specific binding of immune regulators [15]. As part of the latter, the ability to bind factor-H (fH) at the bacterial cell surface and so exploit the host's own protection against the complement response is an essential virulence factor to establish infection and is thought to determine host specificity [16]. Adhesion, invasion and immune evasion is mediated primarily by a variety of surface lipoproteins and membrane spanning β-barrels. Other notable surface proteins include the integrin-binding BBB07 [17], the α-helical P13 channel protein [18,19] and the BesABC (*Borrelia* efflux system proteins A, B and C) efflux pump [20,21].

Lipoproteins are readily identified by their characteristic signal sequence, consisting of a positively charged N-region, hydrophobic H-region and a lipobox terminating with a single cysteine amino acid [22]. Genome data [23,24] has shown *Borrelia* has ∼105 such proteins, many of which have been studied in detail and have been shown to bind to a wide range of host extracellular matrix proteins [25], immune regulators and cell surface receptors [15].

β-Barrel membrane-spanning proteins pose a significant challenge to prediction methods because they are characterized by short (<10 amino acids) membrane-spanning strands of alternate hydrophobic residues interspersed by highly variable loop regions [26]. The sequence conservation between orthologues can also be very low. Consequently and probably also due to their relative scarcity on the outer membrane (OM), very few β-barrel membrane-spanning proteins have been identified in *Borrelia*. Those that have been identified, BesC (BB0142) [20], dicarboxylate-specific porin A (DipA) (BB0418) [27], P66 (BB0603, Oms66) [28,29] and BamA (barrel assembly machinery; BB0795) [30] are all predicted to form 16–24-stranded porin-type barrels. The smaller barrel-types with 8–10 strands, commonly found in other Gram-negative bacteria have so far remained undetected in *Borrelia*.

Highly conserved among the *Proteobacteria*, the outer membrane protein A (OmpA)-like transmembrane (TM) domain, defined by Pfam family PF01389, consists of eight membrane-spanning antiparallel β-strands with four large extracellular loops and three short periplasmic turns [26]. High-resolution structural data are available for several members of this family with a range of different functions [31–34]. This structural data confirmed that the TM region is highly conserved and functional diversity is mainly due to variations in extracellular loop regions. The prototypical *Escherichia coli* OmpA functions as a non-specific diffusion channel and with the addition of a C-terminal peptidoglycan-binding domain, contributes to the maintenance of cell shape and structural integrity of the OM [35].

In addition to these physiological functions, numerous members of this family are known virulence factors including *E. coli* OmpA [36,37], *Cronobacter sakazakii* OmpA [38], *Salmonella typhimurium* Rck and PagC [39,40] and *Yersinia pestis* Ail [31]. These proteins contribute to pathogenicity by binding to a range of host factors mediating adhesion, invasion and resistance to the complement response.

To date, the OmpA-like TM domain has not been identified in *Borrelia*. Considering the similarities between the membrane structure of *Borrelia* and that of the *Proteobacteria*, we considered it likely that the OmpA-like TM domain would be conserved in both. To this aim we conducted PSI-BLAST searches of the NCBI protein database against the available *B. burgdorferi* proteomes using a variety of OmpA sequences as search targets. Although larger β-barrel proteins such as BamA (BB0795) were found, no proteins matching the criteria of an OmpA-like domain could be identified. Consequently, we used a profile hidden Markov model (HMM) search strategy, which enables sequences with more remote homology to be detected [41]. Four homologous proteins were identified, all encoded by chromosomal genes and highly conserved between different species of *Borrelia*. Analysis of signal sequences and bioinformatic analysis suggest that all four proteins adopt similar topology as eight-stranded membrane-spanning β-barrels. As the most common cause of EM in Europe, we chose the *B. afzelii* homologue BAPKO_0422 for further experimental work.

In the following work, we describe the production of a recombinant version of BAPKO_0422 consisting of the predicted membrane-spanning domain (no signal sequence) in the *E. coli* expression system. Furthermore an affinity ligand-binding immunoblot (ALBI) assay demonstrates that BAPKO_0422 binds specifically to human fH. Finally, data from SAXS, CD and phase partitioning demonstrate that BAPKO_0422 adopts a compact peanut-shaped structure rich in β-strand, consistent with a membrane-spanning β-barrel topology.

## MATERIALS AND METHODS

### Identification of *Borrelia* OmpA-like domains

Protein sequences of all available OmpA-like TM domains were obtained from the Pfam database family PF01389 [42]. Sequences with greater than 90% or 40% similarity were removed using the Decrease Redundancy program (ExPASy) to generate two lists, which were then submitted to JACOP for classification [43]. Three families were identified corresponding to OmpA, OmpX and OmpW. Sequences corresponding to each family were aligned using ClustalW2 (EBI) and used to build a profile HMM using HMMER (http://hmmer.janelia.org/). This generated six profile HMMs, which were then used to search the genomes of *B. afzelii*, *B. burgdorferi* and *B. garinii* to generate a list of potential OmpA-like proteins. The top scoring hits were filtered based on the presence of a signal sequence predicted using Signal P 4.1 [44] and fold prediction using the Fold and Function Assignment System server (FFAS03) [45]. The procedure identified two proteins BB0027 and BAPKO_0422 matching all criteria. A PSI-BLAST search was then conducted to identify homologous proteins in *B. afzelii*, *B. burgdorferi* and *B. garinii*. Topology predictions were conducted using the PRED-TMBB web server [46] using the mature sequences of all homologues identified above (minus signal peptide).

### Homology modelling

Homology models were generated using Modeller [47] based on co-ordinates from PDB accession codes 1BXW, 1P4T, 1QJ8, 1QJP, 1THQ, 2ERV, 2F1T, 2K0L, 2X27, 3DZM, 3GP6 and 3QRA. A structure-based multiple sequence alignment was generated using the MatchMaker function in the UCSF Chimera package [48] with default settings. This was manually adjusted to minimize sequence gaps in loop regions. Sequences for the putative *Borrelia* OmpA-like TM domains were then added to this alignment and manually adjusted to match the topology and β-strand positioning as predicted by PRED-TMBB. Multiple models were then generated using Modeller. Theoretical radius of gyration (*R*_g_) values, based on the centre of mass, were calculated using the UCSF Chimera package [48].

### Cloning of BAPKO_0422^20–201^

The coding region for amino acids 20–201 of BAPKO_0422 (European Nucleotide Archive accession number ABH01676.1) was amplified by PCR using the forward primer 5′-GCA-TGGATCCGCAATCAAAAAGCAAAACTAT-3′ and reverse primer 5′-ATCGAAGCTTTCATTTATTCTCCATTATATATA-3′ with the addition of BamH1 and HindIII restriction sites (underlined). This was ligated into the pET-47b(+) expression vector (Novagen) and confirmed by DNA sequencing. PCR primers were synthesized by Eurofins MWG Operon.

### Recombinant expression and purification of BAPKO_0422^20–201^

Protein expression in Rosetta™(DE3)pLysS cells (Merck Millipore) was induced by the addition of 1 mM IPTG to a 3 l of culture of cells with a *D* of 0.6. After 3 h, cells were harvested by centrifugation and lysed on ice by pulsed sonication in 0.3 M NaCl, 50 mM Tris/HCl, pH 8.0. The lysate was incubated with DNase before removal of the soluble fraction by centrifugation (20000 ***g*** for 30 min at 4°C) and the pellet prepared for solubilization by means of several preparative washes (0.3 M NaCl, 50 mM Tris/HCl, 10 mM EDTA, 1 mM DTT, 0.1% Triton-X-100, pH 8.0). The washed inclusion body was then solubilized in 8 M urea, 0.3 M NaCl, 50 mM Tris/HCl, pH 8.0, overnight and clarified by centrifugation at 16000 ***g*** for 30 min.

Protein was loaded on to a HisTrap HP 5 ml column (GE Healthcare) in 8 M urea, 50 mM Tris/HCl, 0.3 M NaCl at pH 8. On-column refolding was performed by the use of a linear gradient into 1 M urea, 50 mM Tris base, 0.3 M NaCl, 0.1% *N*,*N*-dimethyldodecylamine-*N*-oxide (DDAO) at pH 8.0 over 60 ml at a flow rate of 0.5 ml/min. Refolded BAPKO_0422^20–201^ was then subjected to 10 column volumes of wash buffer, 1 M urea, 50 mM Tris/HCl, 1 M NaCl, 50 mM imidazole, pH 8.0, before being eluted by a linear gradient of 50 mM Tris/HCl, 0.3 M NaCl, 0.3 M imidazole, 0.1% DDAO at pH 8.0.

The N-terminal 6× His-tag was removed enzymatically using the HRV-3 (human rhinovirus-3) protease (Sigma–Aldrich). Purified BAPKO_0422^20–201^ was incubated at room temperature for 48 h in buffer 0.3 M NaCl, 0.1% (w/v) DDAO, 50 mM Tris/HCl, pH 8.0. The reaction mixture then was passed through a HisTrap HP 5-ml column (GE Healthcare) to remove the 6× His-tagged HRV-3C and any uncleaved protein.

Protein was concentrated using a 10 NMWL Amicon Ultra-15 centrifugal filter unit (Merck Millipore), centrifuged at 16000 ***g*** for 20 min at 10°C and the protein concentration measured by spectroscopy and Bradford analysis.

### Size exclusion chromatography of BAPKO_0422^20–201^

Following initial purification, BAPKO_0422^20–201^ was subjected to gel filtration and was applied to a Superdex 75 10/300 column at a flow rate of 0.5 ml/min in 0.3 M NaCl, 50 mM Tris/HCl, 0.1% DDAO at pH 8. Peak fractions containing BAPKO_0422^20–201^ were pooled and concentrated. BSA, ovalbumin, ribonuclease A and vitamin B12 were prepared in the same buffer and used as size exclusion chromatography (SEC) standards.

### Affinity ligand-binding immunoblot assays

ALBI assays were performed to detect specific binding to human fH. BAPKO_0422^20–201^ (10 μg) was subjected to native-PAGE alongside human fH (HC2130, Hycult Biotech) (0.5 μg) as a positive control. Negative controls consisted of BSA (10 μg, Sigma–Aldrich) and recombinant superoxide dismutase A (SodA; 10 μg) from *B. burgdorferi* produced from the same vector and subjected to the same purification protocol. All incubations were carried out at 4°C. Proteins were immunoblotted on to PVDF membrane and blocked in 5 % milk powder made up in TBS. Following three 5-min washes with TBS-Tween (0.05 %), the membrane was incubated with human fH at a concentration of 73 μg/ml in TBS for 14 h with gentle agitation. This concentration is approximately 3-fold lower than the mean concentration of fH in human blood (233–269 μg/ml) [49]. The membrane was further washed, as described above, before incubation with the primary antibody for 1 h (1:1000 of mouse monoclonal anti-Human fH, Abcam). Following washing as above, the membrane was then subjected to the secondary antibody for 1 h (1:5000 of Goat Anti-Mouse IgG H&L, Alexa Fluor®680, Abcam). The blot was then visualized on a LI-COR Odyssey infrared imaging device by measuring emission at 700 nm. A negative control blot was carried out alongside the experimental assay. In the latter case, human fH was replaced with TBS for the 14-h incubation.

### CD spectroscopy

CD data were collected from a solution of 0.33 mg/ml BAPKO_0422^20–201^ in 0.1 % (w/v) DDAO, 0.3 M NaCl, 30 mM Tris/HCl, pH 8.0, on a Jasco J-810 Spectropolarimeter. Measurements were taken in duplicate over a wavelength range of 195–260 nm before background subtraction using a dialysis matched buffer. Data were processed using the DichroWeb server using the algorithm CDSSTR and reference sets 4 and 7.

### Phase partitioning

Phase partitioning was conducted according to published methods [50]. Control proteins haemoglobin and lysozyme were purchased from Sigma–Aldrich. *Arabidopsis thaliana* voltage-dependent anion channel 1 (AtVDAC1) was refolded and purified according to published methods [51]. All temperature incubations were for a period of 3 min. Protein solutions were made up to 1 mg/ml in 10 mM Tris/HCl, pH 7.4, 150 mM NaCl, 1% Triton X-114 at 0°C. A 200 μl of sucrose cushion was prepared consisting of 6% (w/v) sucrose, 10 mM Tris/HCl, pH 7.4, 150 mM NaCl and 0.06% Triton X-114. Fifty microlitres of protein solution was overlaid on the sucrose cushion and incubated at 30°C before centrifugation at 300 ***g*** for 3 min. The upper aqueous layer (70 μl) was removed and additional Triton X-114 was added to 0.5 % concentration. After incubation at 0°C and gentle mixing, this aqueous phase was overlaid on the same sucrose cushion, incubated at 30°C and then centrifuged at 300 ***g*** for 3 min to pellet the detergent phase. The upper aqueous phase was removed and received an additional 2% Triton X-114. This was then cooled to 0°C, mixed, then incubated at 30°C before a final centrifugation at 300 ***g*** for 3 min (pellet discarded). The supernatant (aqueous phase) was then analysed by SDS/PAGE alongside the previous detergent phase.

### SAXS

SAXS data were acquired using a Bruker Nanostar from solutions of BAPKO_0422^20–201^ in 0.1 % (w/v) DDAO, 0.3 M NaCl, 50 mM Tris/HCl, pH 8.0. Protein samples of ∼100 μl were sealed in a 1.5 mm bore quartz glass capillary and the sample chamber was evacuated to minimize background scattering. The sample to detector distance was 106.85 cm. The *s*-axis and beam centre were calibrated using the scattering pattern of silver-behenate salt (*d*-spacing=5.84 nm). The momentum transfer was defined as *s*=4πsin(θ)/λ. Although detergent solutions exhibit scattering similar to that of water, proper background subtraction is essential to ensure good quality data. In addition, the presence of protein can sequester detergent from the surrounding solution and alter the monomer/micelle equilibrium. For this reason, samples were dialysed against the blank buffer at 4°C for 5 days prior to data collection. Each scattering data set consisted of 10×2400 s exposures. Scattering data were collected from His-tagged BAPKO_0422^20–201^ at 3.3, 4.5 and 5.5 mg/ml and from native BAPKO_0422^20–201^ at 2.5, 3.5 and 4.5 mg/ml. For each sample, the equivalent scattering data collected for dialysis-matched buffer (10×2400 s) was subtracted using Primus software. Equivalent scattering data were also acquired for lysozyme using identical buffers (result not shown).

*R*_g_ values were evaluated using both the Guinier approximation and also in real space from the entire scattering pattern using the indirect transform program GNOM.

*Ab initio* molecular envelopes were calculated using the DAMAVER program suite, part of the ATSAS package [52,53]. DAMMIN generated 20 independent models by simulated annealing (P1 symmetry) using a bead model, which were then aligned and outliers removed before averaging (Supplementary Figures S1 and S2, Supplementary Tables S1 and S2). A filtered model was generated in DAMFILT. Figures were generated in PyMOL (The PyMOL Molecular Graphics System, Version 1.7.4 Schrödinger).

## RESULTS

### Identification and topology prediction of OmpA-like TM domains in *Borrelia*

The sequences of known OmpA-like membrane-spanning domains, defined by Pfam family PF01389 [42], show high conservation in only a small number of key positions, specifically the various residues involved in forming the two aromatic-girdles, several glycine residues and residues involved in ion pair interactions. The aliphatic residues orientated towards the membrane-interior are highly variable between different proteins whereas the loop-regions are exceptionally variable. Because of this low sequence conservation, we employed a sensitive HMM-based search strategy to find potential OmpA-like domains in the genus *Borrelia*. The results revealed four chromosomally encoded proteins, consisting of three closely related paralogous sequences, BAPKO_0422, BAPKO_0423, BAPKO_0591 and the more distantly related BAPKO_0026. An analysis of whole genome sequences of the 35 species in the Lyme-borreliosis group and seven species in the relapsing fever group [54] revealed conserved orthologues of these proteins in all known *Borrelia* species. Ordered locus names from the three main strains known to be pathogenic to humans (*B. afzelii, B. burgdorferi* and *B. garinii*) are shown in Table 1. All the sequences identified are currently annotated as putative uncharacterized proteins. Fold recognition using the FFAS03 server [45] suggested that BAPKO_0026 was more closely related to *E. coli* OmpW, whereas the BAPKO_0422 paralogous group (BAPKO_0422, BAPKO_0423, BAPKO_0591) bore similarities to a range of proteins including the *Neisseria meningitidis* protein NspA and the *E. coli* proteins OmpA and OmpW (Table 1). However, an analysis of sequence similarity and turn/loop lengths with comparison to the *E. coli* homologues did not reveal any consistent features, suggesting the *Borrelial* proteins share the same basic topology as OmpA/OmpW/NspA but are not closely related to any of these proteins in particular.

**Table 1:** Ordered locus names of potential OmpA-like domains identified by HMM/FFAS03/BLAST searches from *B. afzelii* (BAPKO), *B. burgdorferi* (BB) and *B. garinii* (BG) The top hit from FFAS03 searches are all eight-stranded membrane-spanning proteins (either OmpA, OmpW or NspA). All sequences are currently annotated as putative uncharacterized proteins. Signal sequence prediction results are based on the no TM neural network. *Present as monomer in OM vesicles [65]. ^†^fH-binding activity [59]. ^‡^UniProt sequence is 11 amino acids shorter than GenBank sequence, full sequence is shown.

Ordered locus name	GenBank (UniProtKB) Accession codes	No. of amino acids	FFAS03 highest scoring template	Signal sequence prediction (SignalP 4.1)
BAPKO_0026	ABH01291.1 (Q0SPD7)	211	OmpW (2X27)	N
BB_0027	AAC66429.1 (O51058)	212	OmpW (2X27)	Y
BG0027	AAU06886.1 (Q662Y5)	212	OmpW (2X27)	N
BAPKO_0422	ABH01676.1 (Q0SNA2)	201	OmpA (2K0L)	Y
BB_0405*	AAC66795.1 (O51366)	203	OmpW (2X27)	Y
BG0407^†^	AAU07257.1 (Q661L4)	203	OmpW (2X27)	Y
BAPKO_0423	ABH01677.1 (Q0SNA1)	203	OmpA (2K0L)	Y
BB_0406	AAC66794.1 (O51367)	203	OmpA (2K0L)	Y
BG0408	AAU07258.1 (Q661L3)	203	OmpA (2K0L)	Y
BAPKO_0591	ABH01831.1 (G0IQB2^‡^)	181	NspA (1P4T)	Y
BB_0562	AAC66924.1 (O51510)	180	NspA (1P4T)	N
BG0572	AAU07409.1 (Q660W2)	179	NspA (1P4T)	Y

Topology predictions (PRED-TMBB) of the proteins listed in Table 1 match an eight-stranded β-barrel with long extracellular loops and short periplasmic turns (Figures 1 and 2). Both N- and C-termini are predicted to be periplasmic. A consensus topology was generated based on the PRED-TMBB results and manual inspection of conserved of aliphatic residues (shaded grey, Figures 2A and 2B). The topology prediction failed for BB0405 and BG0407 and some manual interpretation was required based on the sequence similarity to other members of the group. Some uncertainty remains about the positioning of the first strand (indicated by dashed lines in Figure 2). The sequence alignment reveals that TM regions are more conserved than loop regions, which vary in sequence and length between the four homologues. Side chains predicted to occupy the barrel interior are in general more conserved than neighbouring residues exposed to the hydrophobic lipid-bilayer, as might be expected considering the constraints imposed by packing inside the barrel.

**Figure 1:**
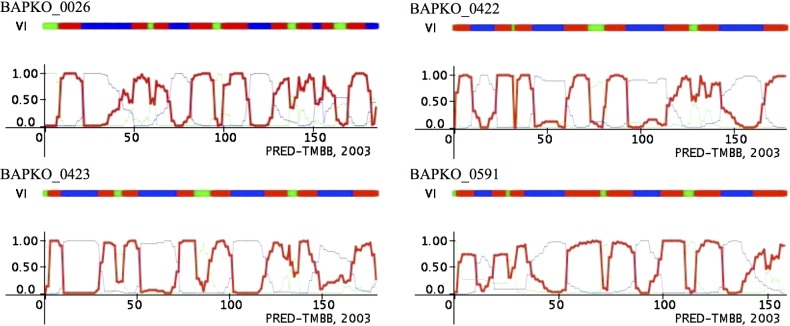
Topology prediction (PRED-TMBB) of ordered loci BAPKO_0026, BAPKO_0422, BAPKO_0423 and BAPKO_0591 Colours indicate regions predicted to be extracellular (blue), periplasmic (green) and TM (red). Sequences are numbered based on the mature protein (no signal sequence).

**Figure 2:**
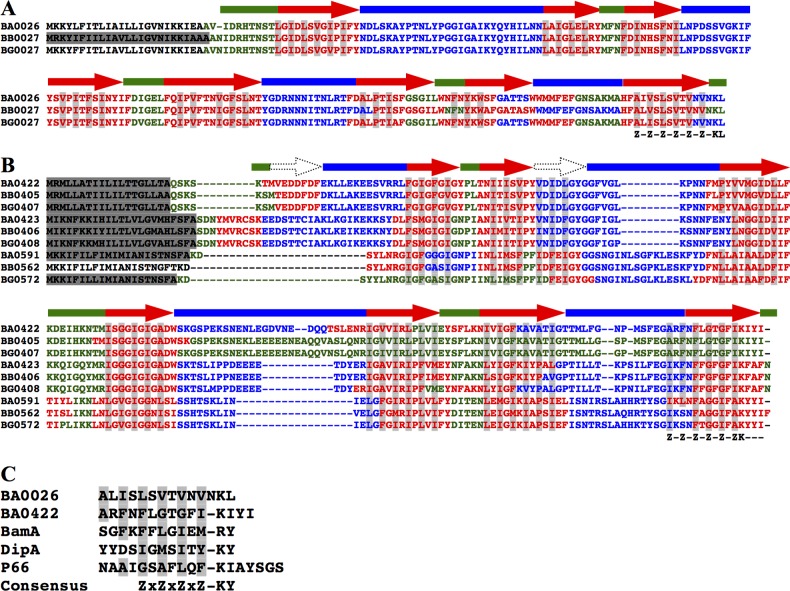
Multiple sequence alignment and consensus topology of the BAPKO_0026 group (A) and the BAPKO_0422 paralogous gene family (B) Sequences from the three main Borrelia sub-species known to infect humans are included (BA, *B. afzelii*; *BB, B. burgdorferi*; and BG, *B. garinii*). Colours indicate predicted topology based on PRED-TMBB results (Figure 1). Non-polar membrane spanning residues are shaded light grey. Predicted N-terminal signal sequences are shaded dark grey (SignalP 4.1, using neural network ‘SignalP-noTM’). Sequences were aligned using ClustalW. A consensus topology prediction is shown above each sequence alignment based on the PRED-TMBB topology and conserved hydrophobic residues. All sequences were analysed for potential stop-transfer sequences using ProtScale. The potential C-terminal signature sequence is indicated by Z-Z-Z-Z-Z-ZK. (**C**) An analysis of C-terminal signature sequences from experimentally confirmed β-barrels BamA (BB0795), DipA (BB0418) and P66 (BB_0603).

Signal sequence prediction (Signal P) [44] suggests that nine out of the 12 homologues listed in Table 1 contain a functional signal sequence (Figures 2A and 2B). Multiple positively charged amino acids are seen in the N-terminal region, a feature consistently found in confirmed signal sequences from both spirochetes and proteobacteria [55,56]. With the exception of BB0562, all sequences have an alanine residue in the predicted −1 position and so are expected to be processed by a type-I signal peptidase.

Inspection of BAPKO_0423, BB0406 and BG0408 shows two cysteine amino acids at positions 31 and 40, indicating potential sites for N-terminal lipidation. However, the potential lipobox is a poor match with previous analysis of spirochetal lipobox sequences [22]. The topology prediction presented in the present study suggest these two cysteine amino acids form part of a small periplasmic region and are therefore likely to be disulfide bonded.

### *Borrelia* OmpA-like domains contain a C-terminal signature sequence

The vast majority of OM β-barrels in Gram-negative bacteria contain a C-terminal signature sequence resembling Z-x-Z-x-Z-x-Y-x-F>, where x represents any amino acid and Z-represents non-polar residues. The terminal residue is invariably a non-polar residue, usually a phenylalanine [57].

The four alternate non-polar residues are well conserved in the C-terminal region of the *Borrelia* OM-proteins (Figures 2A and 2B); however, some differences in the terminal residues are apparent. Only BB0562 terminates with a phenylalanine residue, almost all other sequences terminate with the small non-polar amino acids isoleucine or valine. The putative OM β-barrels identified in the present study contain an invariant lysine residue within four residues of the C-terminus. Following this lysine residue, the C-terminal sequence of the BAPKO_0422 group is rich tyrosine and small non-polar-residues. A comparison was made with the C-terminal regions of other experimentally confirmed *Borrelia* β-barrel proteins BamA, DipA and P66 (Figure 2C). The sequences reveal four alternate non-polar residues followed by a conserved positively charged residue (lysine or arginine).

### Homology model of BAPKO_0422

To allow a more detailed analysis of residue packing within the barrel interior and positioning in the membrane, homology models were generated using Modeller [47] (Figure 3). This modelling benefited from the availability of several high-resolution structures of TM β-barrels from an evolutionary diverse range of bacteria including *E. coli*, *N. meningitidis*, *Y. pestis*, *Pseudomonas aeruginosa* and *Thermus thermophilus.* The four *B. afzelii* homologues shown in Figure 1 were used as target sequences. The models suggest that all four proteins have a vertical height 45–62 Å in the membrane with a width of ∼25 Å (Figure 3). *R*_g_ values based on centre of mass were also calculated and ranged between 17.0 and 19 Å. The modelled β-barrels resemble an inverse micelle, with numerous polar residues and ion-pair interactions forming a tightly packed interior. This is surrounded by an exterior band of aliphatic residues predicted to interact with the non-polar acyl chains of *Borrelia* glycolipids. Aromatic residues are frequently observed at the lipid-water interface in membrane spanning β-barrels of Gram-negative bacteria [26]. Two aromatic girdles are present in the *Borrelia* proteins, allowing the vertical positioning in the membrane to be estimated (dashed line, Figure 3). The distance between the two aromatic girdles (24–27 Å) is consistent with the hydrophobic distance (∼26 Å) of β-barrels from other Gram-negative bacteria [58].

**Figure 3:**
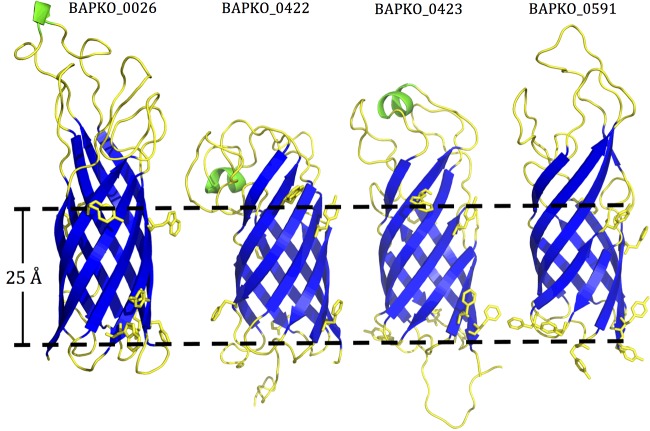
Homology models of BAPKO_0026, BAPKO_0422, BAPKO_0423 and BAPKO_0591 generated using Modeller The predicted secondary structure shows an eight-stranded antiparallel β-barrel (blue). Loops and turns are shown in yellow, α-helices are shown in green. Aromatic-girdle residues are shown as sticks representation. The approximate limits of the aliphatic region are indicated by dashed lines. *R*_g_ values calculated in Chimera: BAPKO_0026 18.9 Å, BAPKO_0422 16.8 Å, BAPKO_0423 18.1 Å, BAPKO_0591 18.4 Å.

### Expression, purification and characterization of recombinant BAPKO_0422

The predicted mature form of BAPKO_0422 (BAPKO_0422^20–201^) was produced in the *E. coli* expression system with a cleavable N-terminal 6× His-tag and purified to homogeneity as described in ‘Materials and Methods’ (Figure 4A). The resultant protein lacked a functional N-terminal signal sequence and so was produced as inclusion bodies, facilitating separation from native *E. coli* membrane proteins. Purified BAPKO_0422^20–201^ readily refolded using a variety of methods including dilution and on-column refolding protocols (see Materials and Methods). Numerous studies have demonstrated that eight-stranded β-barrel membrane proteins can spontaneously refold in lipid bilayers or detergent micelles following either heat- or chemically-induced unfolding [58].

**Figure 4:**
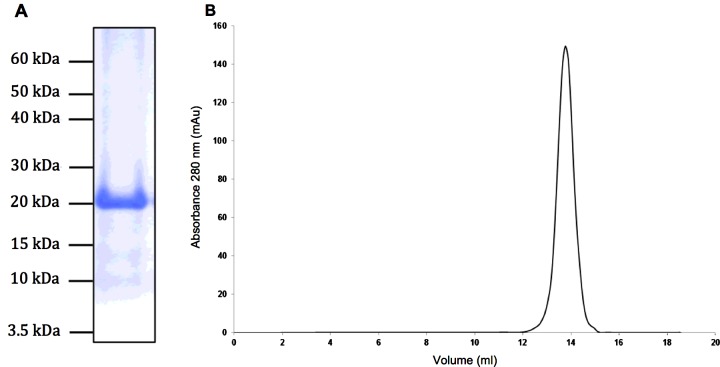
Production and purification of recombinant BAPKO_0422^20–201^ (**A**) SDS/PAGE of purified BAPKO_0422^20–201^ with a single band at ∼23 kDa. (**B**) Size exclusion trace for BAPKO_0422^20–201^. Purified protein was applied on to a Superdex 75 10/300 column at a flow rate of 1 ml/min resulting in a single sharp peak corresponding to an approximate molecular mass of 23 kDa.

The size exclusion chromatogram (Figure 4B) revealed a single peak. The elution volume corresponds to a molecular mass of 23 kDa indicating that BAPKO_0422 forms a monomer under the conditions tested. Protein folding was confirmed by CD, which revealed a classic β-strand-type spectrum with maxima at 196 nm and minima at 216 nm (Figure 5A). The secondary structure analysis predicts 40% β-sheet, 20% turn and 35% unordered.

**Figure 5:**
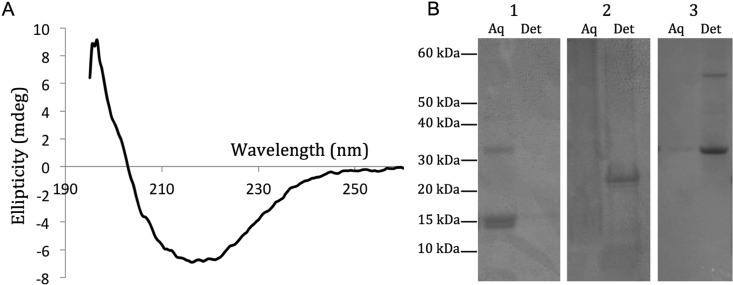
CD (A) and phase partitioning (B) of BAPKO_0422^20–201^ CD data were acquired at 20°C in duplicate at a protein concentration of 0.33 mg/ml in 0.1 % (w/v) DDAO, 0.3 M NaCl, 30 mM Tris/HCl, pH 8, between wavelengths of 195–260 nm. Maxima at 196 nm and minima at 216 nm indicate a structure rich in β-strand. Phase partition experiments were conducted using the non-ionic detergent Triton X-114, allowing separation of hydrophilic proteins (Aq) from amphiphillic detergent-soluble proteins (Det). Haemoglobin (1) was used as an aqueous phase negative control. BAPKO_0422^20–201^(2) partitions to the detergent phase. AtVDAC1 (3) was used as a known detergent-phase positive control.

The relatively low cloud point of Triton X-114 (∼23°C) allows the separation of amphiphilic integral membrane proteins from water-soluble proteins at physiological temperatures. Above the cloud point, aggregation of detergent micelles results in a two-phase system consisting of an aqueous phase and a detergent-rich phase. This detergent phase can be readily isolated by low speed centrifugation. BAPKO_0422 was shown to be amphiphilic, partitioning to the detergent phase (Figure 5B).

### Structural characterization of BAPKO_0422 by SAXS

SAXS data from a solution of a monodisperse protein can provide a measure of the *R*_g_ and overall molecular shape. SAXS data were recorded from both untagged BAPKO_0422^20–201^ (Figure 6A) and His-tagged protein (6His-BAPKO_0422). Inspection of the Guinier region and residuals from linear fitting (Figure 6B) revealed linearity an indication of good quality data with minimal aggregation.

**Figure 6:**
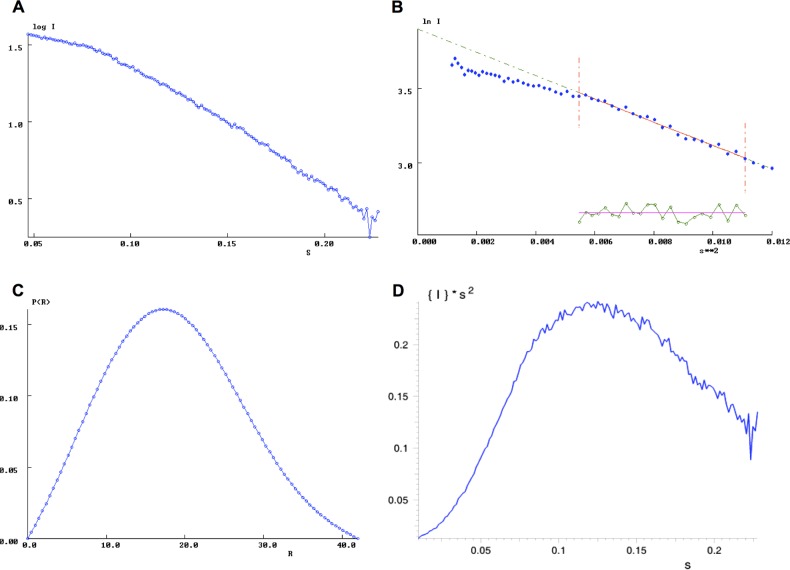
SAXS data of BAPKO_0422^20–201^ Analysis of the scattering data generated from a sample of untagged BAPKO_0422^20–201^ at 3.5 mg/ml in 0.3 M NaCl, 50 mM Tris-base, 0.1 % DDAO, pH 8.0. (**A**) The raw scattering data shown as a log of intensity over the scattering vector (Å^−1^) and generated in Primus following background subtraction. (**B**) Guinier analysis of the background subtracted scattering data. A Guinier approximation was applied manually using Primus as In[I(s)] versus s^2^. The blue data points represent the scattering data, the red line shows the selected Guinier region and the green data illustrates the corresponding residuals. (**C**) The distance distribution function P(r) of BAPKO_0422^20–201^ generated using GNOM with a *D*_max_ of 43 Å. (**D**) Kratky plot [s^2^ × I(s) versus s] generated using Primus. The momentum transfer was defined as s=4π sin(θ)/λ.

Radiation damage may result in sample aggregation or protein misfolding through the course of an experiment. Scattering data were compared from initial and final images with no significant changes observed in either scattering form or calculated *R*_g_ values. Samples were analysed by SDS/PAGE both before and after X-ray exposure and were unchanged. To test for the possibility of concentration dependent effects such as aggregation or inter-particle interference, *R*_g_ values were determined at three concentrations for both His-tagged and native protein (Table 2).

**Table 2:** Calculated *R*_g_ values for BAPKO_0422^20–201^ A summary of *R*_g_ values for untagged and His-tagged BAPKO_0422^20–201^ calculated using various methods. The *R*_g_ was calculated for both samples manually by Gunier approximation and then by Gnom in reciprocal and real space using the full curve.

	Untagged BAPKO_0422^20–201^		
	2.5 mg/ml	3.5 mg/ml	4.5 mg/ml
Guinier *R*_g_ (Å)	13.5±0.15	14.6±0.11	12.5±0.39
Reciprocal space *R*_g_ (Å)	15.7	14.3	14.4
Real space *R*_g_ (Å)	15.7	14.3	14.5
	His-tagged BAPKO_0422^20–201^		
	3.0 mg/ml	4.5 mg/ml	6.0 mg/ml
Guinier *R*_g_ (Å)	14.2±0.13	14.1±0.12	14.0±0.10
Reciprocal space *R*_g_ (Å)	16.8	16.8	16.6
Real space *R*_g_ (Å)	16.8	16.8	16.7

*R*_g_ values calculated by both Guinier analysis and Real Space analysis (Table 2) are comparable to the *R*_g_ calculated from the homology model (Figure 3) indicating that BAPKO_0422 is monomeric under the conditions tested. The pair-distance distribution function [P(r) function] gives information on the shape of a molecule by describing the paired-set of all distances between points in an object (Figure 6C). The single peak is indicative of a single-domain globular protein. The distribution of longest dimensions approaches zero with a concave slope and a *D*_max_ of 43 Å.

A Kratky plot [q^2^I(q) versus q] can be used to distinguish between compact folded structures and unfolded flexible systems. The data show a parabolic curve indicative of a compact globular structure (Figure 6D).

*Ab initio* molecular envelopes of native and His-tagged BAPKO_0422 calculated by simulated annealing reveal similar structures with some minor additional density in the latter (Figure 7). The SAXS envelope is consistent with the dimensions of a β-barrel and the homology model of BAPKO_0422 docked within the envelope shows close agreement (Figure 7).

**Figure 7:**
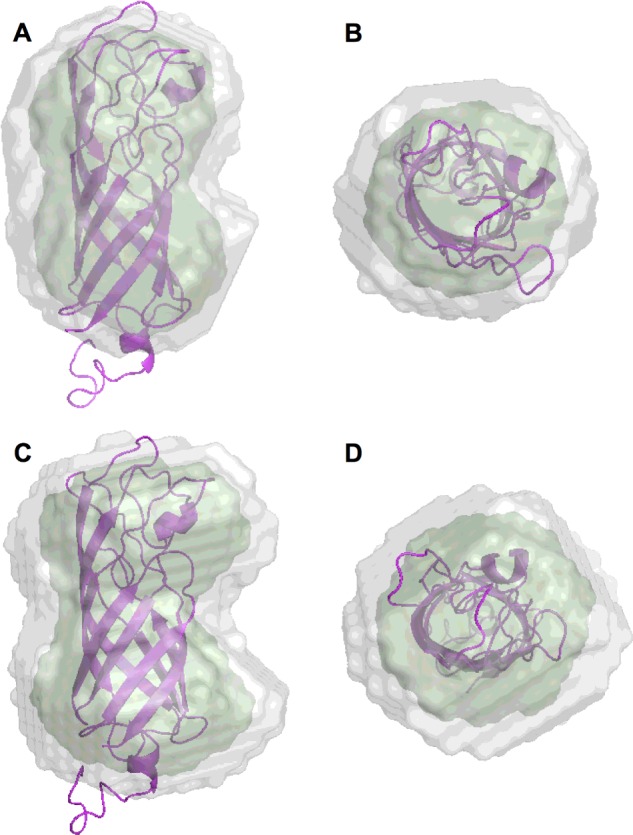
Low-resolution molecular envelopes of BAPKO_0422^20–201^ (A and B) and His-tagged BAPKO_0422^20–201^ (C and D) determined by SAXS Refined and filtered molecular envelopes were generated from 20 independent DAMMIN models (Supplementary Figures S1 and S2). The green surface represents the filtered envelope generated by DAMFILT, the grey envelope is the computed probability map generated by DAMAVER. The homology model of BAPKO_0422^20–201^ is shown as purple cartoon.

### BAPKO_0422 is an fH-binding protein

Numerous studies have revealed the complex differential binding of both human and animal fH by various strains of *Borrelia*, contributing significantly to pathogenicity and host-competence [59,60]. A broad screen of whole cell sonicate from *B. garinii* against human sera followed by *de novo* sequencing identified the hypothetical protein BG0407 (Genbank AAU07257) as a novel fH-binding protein [59]. To test the possibility that the close homologue BAPKO_0422 may also be an fH-binding protein we used a far western blot (ALBI assay). Briefly, immunoblots of BAPKO_0422 along with positive and negative controls were incubated with human fH. Bound fH was detected by a monoclonal Anti-human fH primary antibody, followed by a fluorescent secondary antibody (see Materials and Methods). The results demonstrated that recombinant BAPKO_0422 formed a specific interaction with human fH at a concentration 3-fold lower than that found in human blood (Figure 8).

**Figure 8:**
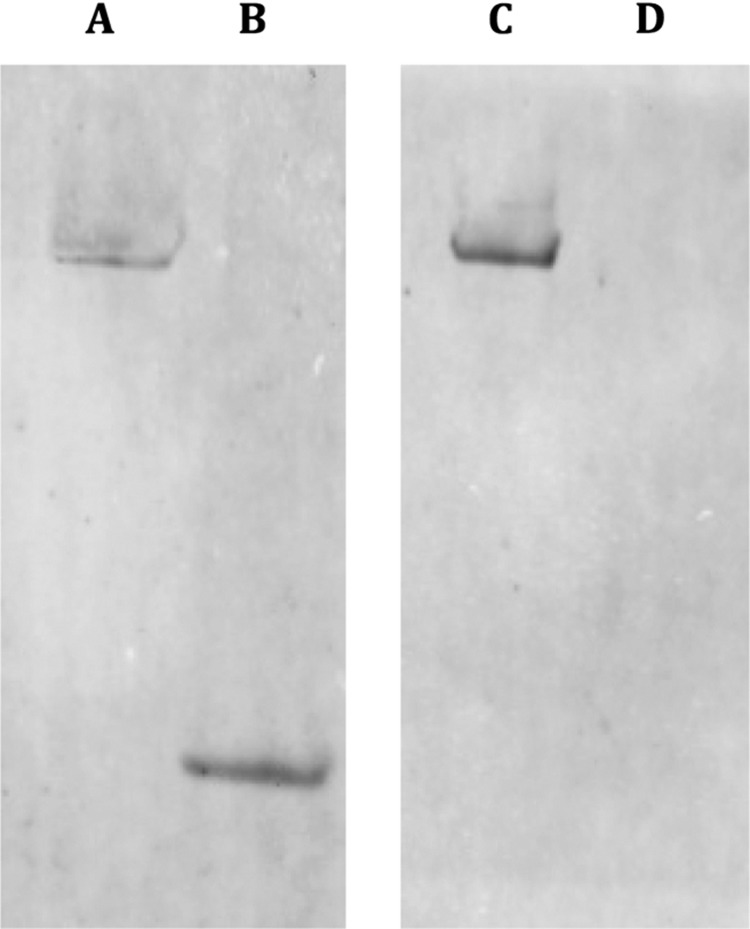
ALBI assay Non-reducing 1D immunoblots of 0.5 μg of human fH as a positive control (lanes A and C) and 10 μg of recombinant BAPKO_0422**^20–201^** (lanes B and D). Blot 1 (A and B) was incubated with human fH (73 μg/ml), whereas blot 2 (C and D) was incubated with TBS buffer. Human fH was detected by a mouse monoclonal anti-human fH primary, followed by a fluorescent goat anti-mouse IgG secondary antibody, as described in ‘Materials and Methods’.

## DISCUSSION

Although the OM of *Borrelia* is distinct from Gram-negative bacteria, the central components of the Sec-dependent secretion pathway and the barrel assembly apparatus involved in localization and insertion of proteins into the OM (BamA, BamB, BamD, Skp), appear to be conserved [30,61]. Genome data are now available for numerous strains of *Borrelia*; however, it remains a significant challenge to identify all potential OM β-barrels due to the inherent difficulties in theoretical prediction. With the exception of the lipoproteins, very few surface exposed proteins have been identified in *Borrelia*. In the present study, we have identified a paralogous group of four *Borrelia* proteins which we predict are eight-stranded membrane-spanning β-barrels. As the most common cause of EM rash in Europe, we chose to study the four *B. afzelii* homologues in more detail. Bioinformatic analyses using a range of prediction methods indicate a topology similar to the well-studied *E. coli* proteins OmpA, OmpX and OmpW. Experimental evidence from CD and a low-resolution SAXS structure of recombinant BAPKO_0422 support this hypothesis.

As recombinant protein was produced from the *E. coli* expression system and purified from inclusion bodies, confirmation of protein refolding is required. The CD data indicate recombinant BAPKO_0422^20–201^ forms extensive secondary structure rich in β-strand. The results are directly comparable to a range of other β-barrels such as OmpW [62], OmpA and OprF [63]. In addition, Kratky plots generated from SAXS data indicate a compact, folded structure. The formation of tertiary structure of many β-barrels proteins can be monitored by the difference in apparent molecular mass between the folded and the unfolded states, as determined by SDS/PAGE. Numerous membrane-spanning β-barrels have been shown to maintain a folded state when solubilized in SDS at room temperature, but once denatured by boiling will remain in an unfolded state [64]. Therefore, gel-shift assays are conducted by comparing boiled and unboiled samples. Gel-shift assays were conducted using recombinant BAPKO_0422; however, the protein remained unmodified by heat (results not shown). This result is consistent with studies on the 24-stranded P66, which was also not modified by heat [28] and is perhaps indicative of a general feature of OMPs in *Borrelia*.

The *ab initio* molecular envelope of BAPKO_0422 determined by SAXS reveals a peanut shaped structure with dimensions 25×45 Å. This structure is consistent with a monomeric eight-stranded β-barrel and suggests a multimeric porin-type structure is unlikely. This is consistent with data on the orthologous protein from *B. burgdorferi* BB0405, which was shown to be monomeric while not making significant interactions with other major OM-proteins [65].

An analysis of N-terminal signal sequences of the BAPKO_0422 family of proteins revealed that the majority are predicted to have a functional signal sequence and so may enter the Sec-dependent secretory pathway for translocation across the inner membrane. In common with OM-proteins from other Gram-negative bacteria the *Borrelia* proteins identified in Table 1 are devoid of long hydrophobic stretches, precluding lateral transfer from the translocase to the inner membrane [66]. Some differences in N-terminal sequence between the three sub-species of *Borrelia* are seen. The N-terminal sequence of the *B. burgdorferi* protein BB0562 may not be recognized by signal peptidase I as it lacks a small non-polar residue normally present at the −1 position. Additionally BA0026 and BG0027 are not predicted to have functional signal sequences, whereas the close orthologue BB0027 does (Figure 2). These differences in predicted signal sequence may be due to subtle differences between the spirochetal signal peptides compared with those of the signal P training set. Alternatively, *Borrelia* is known to have three type-I signal peptidases compared with the single protein in *E. coli* [67]. This allows for the possibility of concerted divergence of signal peptides along with their corresponding peptidase resulting in a wider range of functional signal sequences in *Borrelia* compared with *E. coli*.

The homology models and topology predictions clearly show that the Borrelia OmpA-like domains listed in Table 1 are devoid of any C-terminal domains. The C-terminal residues are therefore predicted to form the terminal strand of the β-barrel and as such are expected to contain the highly conserved C-terminal signature sequence motif [68]. This 10-residue signature sequence consists of three alternating non-polar residues at positions −9, −7 and −5 from the C-terminus. These side chains make contact with the aliphatic region of the membrane. The −3 residue is usually a tyrosine residue that forms part of the aromatic girdle occupying the interface between polar and non-polar environments [57]. The C-terminal residue is usually a phenylalanine but may occasionally be substituted with other aromatic amino acids or more rarely other non-polar amino acids. Although not essential for proper processing, the aromatic nature of the C-terminal residue enhances processing by the BamA apparatus, facilitating barrel assembly in the OM [57,69]. Variations in the C-terminal signature sequence and their recognition by BamA have been shown to be species specific, in particular, many Proteobacterial OMPs have a positively charged residue in the penultimate position, whereas this is never observed in *E. coli* [70]. A C-terminal signature sequence is apparent among the BAPKO_0422 family and BA0026, both consisting of a series of six alternative non-polar residues, a single lysine residue and a small polar terminal motif (Figure 2). With the exception of BB0562, the most notable exception to the standard proteobacterial C-terminal sequence is the absence of a C-terminal phenylalanine residue. Instead the sequences terminate with either the small non-polar residues leucine, isoleucine or the polar asparagine. In addition, a number of tyrosine residues are also present in the final three residues. The differences observed in the present study may be suggestive of a *Borrelia*-specific C-terminal signature sequence. In order to confirm this, we analysed the C-terminal strands of the small number of experimentally confirmed β-barrels in *Borrelia*. DipA has a C-terminal signature sequence reminiscent of the BAKPO_0422 family, terminating with K-Y. In addition, the barrel assembly apparatus BamA, is itself a β-barrel and the last membrane-spanning strand terminates with R-Y. The terminal strand of the OM porin P66 also contains the conserved positive lysine residue and terminates with the polar sequence S-G-S. We therefore propose that these membrane-spanning β-barrels contain a *Borrelia-*specific C-terminal signature sequence and this is recognized efficiently by the *Borrelia* BamA apparatus. This sequence resembles Z-x-Z-x-Z-x-Z-[KR]-[ILNY], where Z represents a small non-polar residue, x can be any amino acid. A conserved positively charged residue is present, but is not necessarily the penultimate residue. The terminal residue appears to be variable and is rarely a phenylalanine residue. An analysis of *Borrelia* proteins identified as potential β-barrels by the TMBB-DataBase [71] reveals a range of C-terminal residues including isoleucine, valine, lysine, asparagine and tyrosine.

A literature search for homologues of BAPKO_0422 from other *Borrelia* subspecies revealed insights into possible functions and cellular localization. The *B. burgdorferi* homologue BB0405 was shown to be surface exposed, present in OM vesicles [65] and expressed in conditions representative of both tick and host environments [72]. Another homologue, BB0407 (Genbank AAU07257) was identified from a screen of proteins with fH-binding activity suggesting a possible role in virulence [59]. This fH-binding activity led us to investigate the fH binding of BAPKO_0422, which we confirmed using an ALBI assay. These results and the high sequence similarity suggest that the orthologous group BAPKO_0422, BG0407 and BB0405 are expressed in the mammalian host, exposed at the cell surface and bind to human fH.

In summary, the data presented suggest that BAPKO_0422 forms a membrane-spanning β-barrel. The topology prediction matches the OmpA-membrane spanning domain defined by Pfam family PF01389, consisting of eight membrane-spanning β-strands linked by short periplasmic turns and longer extracellular loops. For the first time, this extends the species distribution of the PF01389 domain to include members of the *Spirochete* phylum. The orthologous group consisting of BAPKO_0422, BB0405 and BG0407 are proposed to play a role in virulence by binding to host fH, therefore abrogating the host complement response and reducing antigenicity of surface exposed loops. Further work is required to establish the host-specific fH-binding activity of the remaining homologues and to determine any other physiological functions of these proteins in *Borrelia.* Surface exposed epitopes may enhance recombinant immunoblots currently used in diagnostic tests for Lyme disease.

## Abbreviations

ALBI: affinity ligand-binding immunoblot
AtVDAC1: *Arabidopsis thaliana* voltage-dependent anion channel 1
BamA: barrel assembly machinery
BB: *Borrelia burgdorferi*
BG: *Borrelia garinii*
BAPKO: *Borrelia afzelii* strain PKO
DDAO: *N*,*N*-dimethyldodecylamine-*N*-oxide
DipA: dicarboxylate-specific porin A
EM: erythema migrans
fH: factor H
HMM: hidden Markov model
OM: outer membrane
OmpA: outer membrane protein A
*R*_g_: radius of gyration
TM: transmembrane
